# Ischiofemoral impingement syndrome, an unusual entity of hip pain: A case report and literature review

**DOI:** 10.1016/j.radcr.2024.12.049

**Published:** 2025-01-18

**Authors:** Praveen K. Sharma, Sam Raja, Stany Jerosha, Nivashini GR

**Affiliations:** Department of Radiology, Saveetha Medical College and Hospital, Saveetha Institute of Medical and Technical Sciences (SIMATS), Saveetha University, Chennai, Tamil Nadu 602105, India

**Keywords:** Ischium, Femur, Arthralgia, Magnetic resonance imaging, Anti-inflammatory agents, Exercise therapy

## Abstract

Ischiofemoral impingement syndrome (IFIS) is a rare condition that can cause significant hip pain, often linked to past injuries or surgeries. We present a case of a 33-year-old male who has persistent pain in both hips, radiating down his legs and experiencing a snapping sensation without any history of trauma or surgery. Magnetic resonance imaging (MRI) revealed swelling in the quadratus femoris muscle and reduced space between his ischium and femur, typical signs of IFIS. Instead of opting for surgery, the 33-year-old male managed with anti-inflammatory medications, physical therapy, and a targeted exercise program. The pain gradually subsided, and the 33-year-old male regained complete movement in the hip. This case is noteworthy because it shows that non-surgical treatments can successfully manage IFIS, even in the absence of trauma. This case emphasizes the need to consider IFIS when diagnosing unexplained hip pain.

## Introduction

Ischiofemoral impingement syndrome (IFIS) is a rare but increasingly recognized cause of hip and groin pain, characterized by an abnormal narrowing of the space between the ischium and the lesser trochanter of the femur [1]. This narrowing can cause the quadratus femoris muscle to compress, resulting in muscle edema and persistent discomfort [2]. Although initially described in post-surgical or post-traumatic patients, IFIS is now known to occur in individuals without any prior hip trauma or surgery. The illness frequently presents with nonspecific symptoms, making diagnosis difficult, especially without an apparent injury history [3,4]. Magnetic resonance imaging (MRI) is the best way to diagnose IFIS because it can show the usual signs of a narrowed ischiofemoral and quadratus femoris space, which causes quadratus femoris muscle edema [5]. Although less sensitive, radiographs may show a narrowing of the ischiofemoral space or cystic changes in chronic cases. The treatment of IFIS ranges from conservative therapy, such as anti-inflammatory medications and physiotherapy, to surgical management. This article examines the symptoms, diagnosis, and available treatments for IFIS and centers on a single case of IFIS without trauma history, emphasizing the potential for a non-surgical, successful outcome [6].

## Case presentation

A 33-year-old male presented to the emergency room (ER) with bilateral progressive hip pain for 2 years, which was insidious in onset and persistent even at rest. The pain was radiating to both lower extremities. The patient denied any injury and had no significant medical or surgical history. During the physical examination, both internal and external rotation exacerbated the pain, but there was no restriction of movement in the bilateral hip joints. At the same time, ischiofemoral impingement is primarily considered a mechanical issue. We perform laboratory tests to rule out systemic conditions or other contributing factors.

**Fig. 1:**
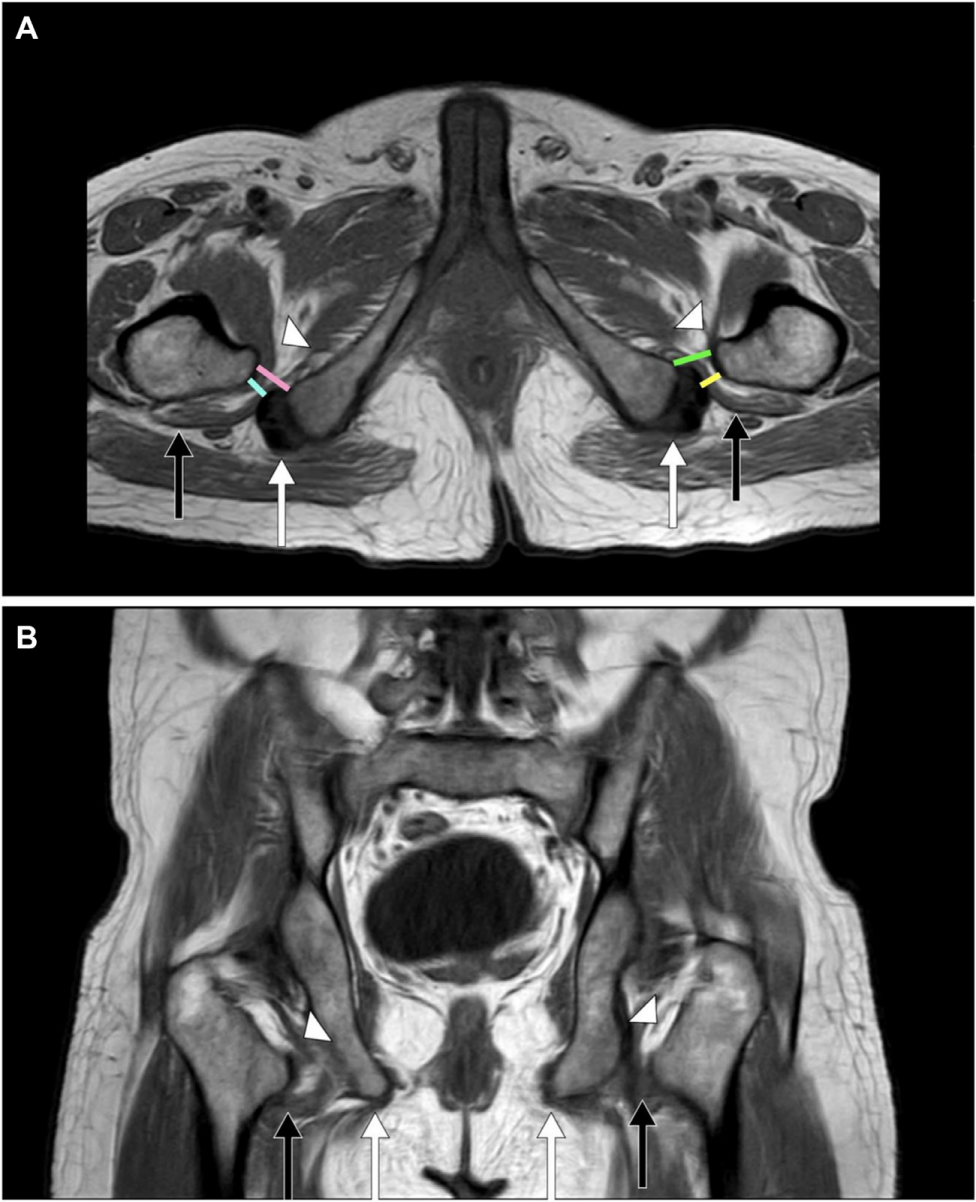
A 33-year-old male presented to the emergency room (ER) with bilateral progressive hip pain for 2 years, which was insidious in onset and persistent even at rest. *Magnetic resonance imaging (MRI) of the hips: T1-weighted (T1W) (A) Axial and (B) Coronal sections* shows a mild decrease in the right ischiofemoral space and quadratus femoris space, measuring 11 mm and 10 mm in the distance respectively and a mild decrease in the left ischiofemoral space and quadratus femoris space, measuring 12 mm and 10 mm in the distance respectively with iliopsoas tendons *(white arrow heads)*, quadratus femoris muscles *(short black arrows),* and hamstring tendons *(short white arrows).* Note: right ischiofemoral space *(pink line),* right quadratus femoris space *(cyan line),* left ischiofemoral space *(green line)*, and left quadratus femoris space *(yellow line).*

**Fig. 2:**
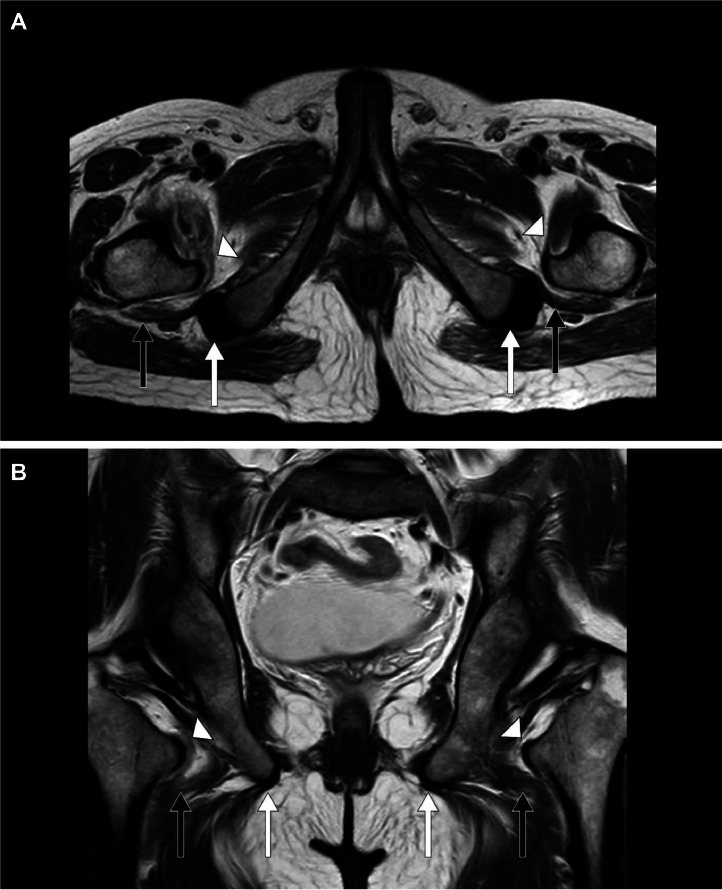
A 33-year-old male presented to the emergency room (ER) with bilateral progressive hip pain for 2 years, which was insidious in onset and persistent even at rest. *Magnetic resonance imaging (MRI) of the hips: T2-weighted (T2W) (A) Axial and (B) Coronal sections* shows a mild decrease in the right ischiofemoral space and quadratus femoris space, measuring 11 mm and 10 mm in the distance respectively and a mild decrease in the left ischiofemoral space and quadratus femoris space, measuring 12 mm and 10 mm in the distance respectively with iliopsoas tendons *(white arrow heads)*, quadratus femoris muscles *(short black arrows),* and hamstring tendons *(short white arrows)*.

**Fig. 3:**
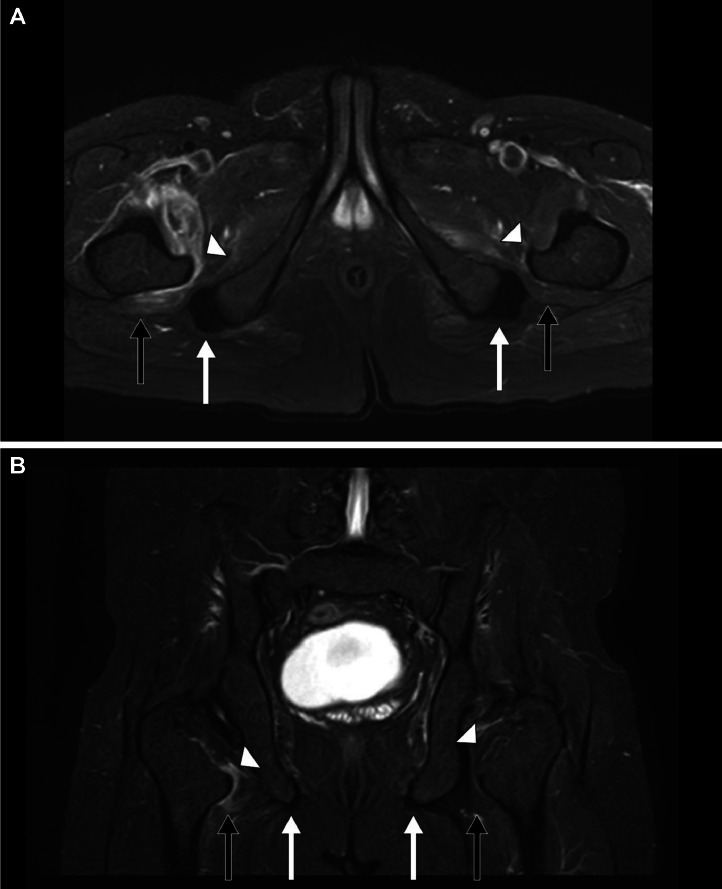
A 33-year-old male presented to the emergency room (ER) with bilateral progressive hip pain for 2 years, which was insidious in onset and persistent even at rest. *Magnetic resonance imaging (MRI) of the hips: Short tau inversion recovery (STIR) (A) Axial and (B) Coronal sections* shows hyperintensities (edema) in the bilateral iliopsoas muscles *(white arrow heads)*, bilateral quadratus femoris muscles *(short black arrows),* and normal bilateral hamstring tendons *(short white arrows).*

**Fig. 4:**
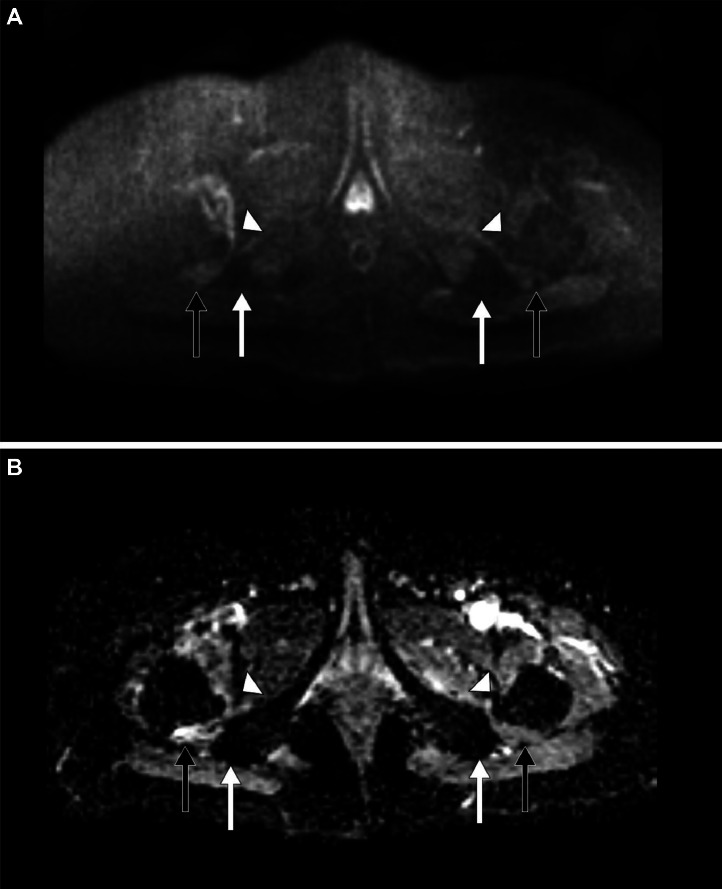
A 33-year-old male presented to the emergency room (ER) with bilateral progressive hip pain for 2 years, which was insidious in onset and persistent even at rest. *Magnetic resonance imaging (MRI) of the hips: (A) Diffusion-weighted imaging (DWI) Axial* – no reduced diffusivity, (B) *Apparent diffusion coefficient (ADC) Axial* – no altered signal intensities)} in the bilateral iliopsoas muscles *(white arrow heads)*, bilateral quadratus femoris muscles *(short black arrows),* and normal bilateral hamstring tendons *(short white arrows).*

Magnetic resonance imaging (MRI) of the hips shows T1-weighted (T1 W) and T2-weighted (T2 W) shows a mild decrease in the right ischiofemoral space and quadratus femoris space, measuring 11 mm and 10 mm in the distance respectively and a mild decrease in the left ischiofemoral space and quadratus femoris space, measuring 12 mm and 10 mm in the distance respectively. Short tau inversion recovery (STIR) shows hyperintensities (edema) in the intramuscular and intermuscular of the right and left proximal thigh region (iliopsoas, quadratus femoris muscles), respectively. Diffusion-weighted imaging (DWI) shows no reduced diffusivity, and apparent diffusion coefficient (ADC) shows no altered signal intensities in the right and left proximal thigh region (iliopsoas, quadratus femoris muscles), respectively [Figs. 1A and B, 2A and B, A and 3B, 4A and B].

——- Based on clinical and imaging findings, bilateral ischiofemoral impingement syndrome (IFIS) is considered.

The diagnostic criteria for IFIS include clinical symptoms and imaging findings. The MRI revealed the typical symptoms, such as a decreasing ischiofemoral space, edema in the quadratus femoris muscle, and no other significant hip problems. All of these were present. IFIS was also consistent with the clinical presentation of bilateral hip pain, exacerbated by rotation, and without any history of trauma.

Due to the lack of structural abnormalities, the 33-year-old male received conservative treatment (anti-inflammatory medications) to reduce pain and inflammation and a physical therapy regimen focused on stretching and strengthening exercises for the hip muscles. Specific exercises aimed to improve flexibility and minimize mechanical impingement in the ischiofemoral region.

Follow-up evaluations indicated a marked reduction in pain, especially during hip rotation and weight-bearing activities, with no recurrence of radiating pain to the lower extremities. The 33-year-old male resumed normal daily activities without discomfort, demonstrating the effectiveness of non-surgical management for bilateral IFIS.

## Discussion

Ischiofemoral impingement syndrome (IFIS) is a relatively recent concept in musculoskeletal medicine, emerging as a significant cause of hip pain and dysfunction. This syndrome involves impingement between the lesser trochanter of the femur and the ischial ramus, which can lead to pain, inflammation, and reduced mobility. As our understanding of IFIS evolves, it becomes crucial to delve into its pathophysiology, diagnostic challenges, and management strategies to enhance patient care. This discussion synthesizes current knowledge about IFIS, emphasizing recent findings and clinical insights.

### Etiology and pathophysiology

Mechanical impingement in the ischiofemoral space, defined by the lesser trochanter of the femur and the ischial ramus, characterizes IFIS. This space can become compromised due to anatomical variations or external factors, leading to symptomatic impingement. The principal pathophysiological cause is compression and inflammation of the surrounding soft tissues, which include the quadratus femoris muscle. The L4, L5, and S1 roots form a small branch of the sacral plexus that innervates the quadratus femoris muscle. The nerve to the quadratus femoris muscle comes out of the body at the pelvis through the greater sciatic notch. It then follows an anterior-inferior course to the gemellus and obturator internus. It penetrates the quadratus femoris muscle on its anterior surface, which performs adduction and external hip rotation **[**7]. The narrowing of the ischio-femoral space is a critical feature in IFIS. This narrowing can be due to bony abnormalities or inflammatory changes. When the lesser trochanter impinges on the ischial ramus, it causes mechanical irritation, leading to local inflammation and pain. The inflammatory response can further exacerbate symptoms and contribute to functional impairment.

### Clinical presentation

Hip pain, either localized or radiating to adjacent areas like the posterior thigh or groin, typically presents in patients with IFIS. The pain often worsens with activities involving hip flexion or internal rotation, which can indicate mechanical impingement. Clinical symptoms may also include reduced range of motion and discomfort during activities that place stress on the hip joint. The presentation of IFIS can overlap with other hip pathologies, making accurate diagnosis challenging. Common symptoms shared with other conditions include groin pain, hip stiffness, and decreased mobility [8,9]. As a result, a thorough clinical evaluation is required to distinguish IFIS from other possible causes of hip pain.

### Diagnostic imaging

Accurate diagnosis of Impingement of the Lesser Trochanter on the Ischium (IFIS) relies heavily on advanced imaging techniques, with Magnetic Resonance Imaging (MRI) playing a pivotal role. MRI is invaluable in viewing and distinguishing IFIS from other hip diseases due to its superior soft tissue resolution. Key MRI indicators of IFIS include a reduced space between the femur and ischium and hypertrophy of the quadratus femoris muscle [10]. These features are critical in diagnosing IFIS as they provide clear evidence of mechanical impingement and associated inflammation. Additionally, MRI allows comprehensive visualization of soft tissues, aiding in the assessment of the degree of impingement.

Despite its advantages, diagnosing IFIS using MRI presents certain challenges. Distinguishing IFIS from other hip pathologies with similar presentations, such as piriformis syndrome and lumbar spinal stenosis, can be difficult. These conditions often have overlapping symptoms, making accurate interpretation of MRI findings and clinical correlation essential to avoid misdiagnosis and ensure appropriate treatment.

Comparative studies have consistently affirmed MRI as the preferred imaging modality for diagnosing IFIS. Its ability to provide detailed images of the ischio-femoral space and associated soft tissues makes it superior to other imaging techniques, particularly in detecting the subtle changes associated with IFIS. This diagnostic precision underscores MRI's importance in the evaluation and management of patients with suspected IFIS.

### Differential diagnosis

Impingement of the Lesser Trochanter on the Ischium (IFIS) has a broad differential diagnosis that includes several conditions with overlapping clinical presentations. These conditions must be carefully differentiated through clinical evaluation and imaging studies to ensure accurate diagnosis and appropriate management.

Piriformis syndrome shares similarities with IFIS, as it may present with hip pain and discomfort, often exacerbated by activities involving hip rotation [11]. However, a key difference is that piriformis syndrome typically causes pain radiating along the sciatic nerve due to nerve compression or irritation. MRI findings in piriformis syndrome do not reveal narrowing of the ischiofemoral space or edema of the quadratus femoris muscle, further distinguishing it from IFIS.

Lumbar spinal stenosis is another condition that may mimic IFIS, as it can cause hip and leg pain, with symptoms worsening during specific activities [12]. The distinguishing feature of lumbar spinal stenosis is the presence of neurological symptoms, such as leg pain radiating down the limb, often accompanied by relief during forward flexion. MRI findings typically reveal narrowing of the spinal canal or compression of nerve roots, with no changes in the ischiofemoral space, differentiating it from IFIS.

A hip labral tear may also result in hip pain and a decreased range of motion, similar to IFIS [13]. However, unlike IFIS, a labral tear affects the anterior or lateral aspects of the hip rather than the posterior thigh. MRI or MR arthrography is instrumental in diagnosing labral tears by identifying the tear itself, without any evidence of ischiofemoral space narrowing.

Osteoarthritis (OA) of the hip can present with hip pain, stiffness, and reduced range of motion, resembling some features of IFIS [14]. However, OA typically produces a more diffuse pain pattern and joint deformity. MRI findings in OA include joint space narrowing, osteophyte formation, and cartilage loss, which are distinct from the ischiofemoral space changes seen in IFIS.

Tendinopathies, such as those involving the iliopsoas or gluteal tendons, can cause localized hip pain and discomfort that may be exacerbated by movement [15]. These conditions differ from IFIS as they are localized to specific tendons rather than generalized impingement pain. MRI findings in tendinopathies typically demonstrate tendon abnormalities, such as tendinopathy or tears, without any narrowing of the ischiofemoral space or associated quadratus femoris edema.

By systematically evaluating clinical features and correlating them with imaging findings, these differential diagnoses can be distinguished from IFIS, allowing for precise diagnosis and targeted treatment.

### Management strategies

Depending on the severity of symptoms and the patient's response to initial treatments, management strategies for IFIS can be either conservative or surgical [16].

• *Conservative Management:* Conservative strategies for IFIS typically include physical therapy, activity modification, and anti-inflammatory medications [17]. Physical treatment seeks to strengthen hip muscles, increase flexibility, and relieve mechanical stress on the ischiofemoral space. Activity adjustment involves avoiding activities that worsen symptoms, while anti-inflammatory drugs can assist in managing pain and inflammation.
• *Surgical Intervention:* When conservative treatment fails, surgical intervention becomes viable. Surgical options generally involve directly addressing the impingement, which may include debridement or modification of the bony structures [18]. The surgical procedure choice depends on the severity of the impingement and the specific anatomical factors involved.

### Postoperative considerations

Postoperative management of IFIS includes monitoring for symptom recurrence and evaluating the intervention's success. Follow-up imaging frequently uses MRI to assess the healing process and confirm that the impingement has received sufficient attention. During postoperative rehabilitation, physical therapy restores function and prevents symptom recurrence.

## Conclusion

Ischiofemoral impingement syndrome (IFIS) is a rapidly emerging cause of hip pain that can have a practical impact on an individual's quality of life. Despite its relative rarity, IFIS should be considered in the differential diagnosis of hip and groin pain, particularly in patients who present with non-specific symptoms and have no history of trauma or surgery. Advances in imaging, particularly MRI, have been instrumental in improving the diagnosis of IFIS, allowing for earlier detection and more targeted treatment strategies. While conservative management, such as physical therapy and anti-inflammatory drugs, has proven beneficial in many cases, surgical intervention is required for patients who do not respond to nonsurgical treatments. This article's case highlights the significance of timely diagnosis, awareness of IFIS, and the potential for successful outcomes through appropriate management. Future research should focus on refining diagnostic criteria, exploring the underlying pathophysiology, and evaluating the long-term consequences of various treatment modalities to enhance the care of patients with this condition.
